# Remarks on Medical Organization

**Published:** 1852-07

**Authors:** Henry Hartshorne

**Affiliations:** Philadelphia


*Remarks on Medical Organization.* By HENRY HARTSHORNE, M. D., of Philadelphia.

A close attention to the proceedings of the recent National Convention at Richmond, has given rise to some reflections, which may be expressed briefly.

It seems to have become a general opinion, that the precise mode of representation by which the national body has been aggregated, is a mere "provisional government," suited to the emergency of its first formation, but inappropriate to its maturity. Several different plans proposed for its modification in 1851 were reported upon this year, by a committee appointed for the purpose; and the subject was then referred to another committee, which submitted a series of definite propositions for amending the constitution. These will come up for adoption or rejection next year. In the meantime, it is of some importance that the profession generally should form correct conclusions on the subject, so as to influence the decision of the Association.

We have no intention of occupying space with a discussion of the whole subject; but stop merely to observe, that all such reforms must be gradual, and that the committee of 1852 acted wisely in preferring, at present, an alteration in the *ratio* of representation of Colleges and Hospitals, to their entire exclusion.

But a topic to which more particular attention may be directed, is that of permanent membership. The propositions submitted by the committee of this year, and adopted as a report by the convention, to be finally acted upon in 1853, embrace a provision for the abolition of all permanent membership; leaving to the Association an interrupted life only,—having no members except the delegates appointed for the particular meeting of each year. Now, to this alteration, there exist many and weighty objections. In the first place, with regard to the permanent members already created, it would seem to be a measure of questionable justice and right, summarily to deprive them of a privilege guaranteed by an original and fundamental regulation of the constitution under which they served. But, waiving this, we may take the ground of an established precedent, and assert that the *onus probandi* rests with the proposers of the innovation, to justify it, if they can. *What harm* is done by the existence of permanent memberships? What good will be effected by their abolition? These are the questions at issue. If they be answered negatively, it must be an error to make the change.

Now, as the permanent members, not delegates, have no vote, it is impossible for them to do any harm, unless by the power of speech, in the meetings of the Association. And the fact of their having this power of speech, need only increase the care and responsibility of each of the bodies sending delegates, that those sent may not be such as to act as either incendiaries or time-killers at any future time. The Association has, moreover, of course, the power to expel members who have proved themselves really unworthy.

But, it has been hinted, that the institution is not republican; that it creates a privileged class; an aristocracy. Not so, by any means;—because these privileges are within the reach of all, in turn, and should be made to rotate amongst all, whose characters and attainments are worthy of a place in the great American fellowship. If there be *any* comparison, where members compete, as must be the case to some extent in every profession and in every class, surely "οι αριστοι" *ought* to be those upon whom the principal choice should fall. The plan of constituting every member of every county society, ipso facto, member of the National Association, and conferring upon each a diploma to that effect, would have this result simply; that it would add one more to the number of formal certificates whose real value is in inverse proportion to their abundance, and which carry but little force in their assertions. Every member of a County Medical Society *is* a member of the great body of American Physicians; but what would be added to the fact by its inscription upon parchment? Or why should this fact interfere with the existence of a special privilege, for a particular purpose, delegated each year to some, though open in turn to all?

But the real argument of importance against the abolition of permanent memberships, is in connection with the appointment of the Special Committees of the Association, upon scientific subjects. How it would narrow the selection and efficiency of these, to carry out the proposed change! It was, indeed, with judgment, proposed this year to extend the service of the Committees on Epidemics to five years, to allow of the accumulation and digestion of more valuable facts. How, then, could this be done, if those appointed delegates one year, cease with that year to be members of the Association, and may happen never to be appointed again? The most cursory observation of the requirements and difficulties connected with these committees, must show the immense advantage of leaving, for their selection, the wider range allowed by the present order of the constitution.

But it is in immediate connection with the business of these committees of observation, that we would beg leave, with great diffidence, to offer a few farther suggestions. Their germ was found in a report read at Richmond, this year, by Worthington Hooker, M. D., of Connecticut, as chairman of a committee on Epidemics. In this was expressed, very clearly, the disappointment of the committee in their attempts to obtain full materials for their report by correspondence. But one or two answers were obtained, if we recollect rightly, out of a large number of letters and circulars addressed to physicians through an extended district. It was hence inferred, that a deficiency or inapplicability exists in the method by which these materials were sought; and a wish was expressed, that some mode of organization might be adopted, by which medical observation could be made more available to the same ends.

It appears to the writer, that nothing more important could be suggested in connection with the business of the National Association. The objects of this great organization of the profession would seem to be chiefly of two kinds: 1. Medico-legislative or ethical,—and 2. Scientific. And, as all medical legislation looks to the application of science as its subject of control, we may consider the advancement, diffusion, and legitimate use of medical knowledge to be the great ends of all professional organization. To *accumulate* and to *concentrate medical observation*, systematically, throughout so large a country as ours, would be one of the greatest benefits it could confer. It affords also excellent opportunities for accomplishing this. It has been attempted, by the appointment of yearly committees, for instance, upon Epidemics. But how has this plan succeeded ? Besides Dr. Hooker's, numerous other reports have asserted, upon the same and other occasions, a large preponderance of disappointment. The question then remains, whether some other plan cannot be devised, by which stimulus, facility, and universality may be given to the collection of data concerning disease, and their centralization ensured for the benefit of the whole country.

For this, it needs only a methodical and determined commencement, originating in the central body, and ramifying throughout its local constituencies. In other departments of science, (meteorology for instance) all are familiar with the extent and success of plans for the accumulation of detailed facts and observations, by the issuing of blank forms and circulars, to be filled up in scattered localities, and all returned to a central generalizing functionary or board. In the Report of the Committee on Hygiene, of the American Medical Association, published in its transactions for 1851, a recommendation, somewhat similar to this, is given, in pursuance of a suggestion from the Smithsonian Institute ; but still very limited in its bearing. This committee "recommend that the blank forms furnished by the Institute should be distributed by the State Medical Societies to their members, with an earnest request that they should make faithful returns of all deaths occurring in their practice. That the returns received by the State Societies should be tabulated and arranged upon a general and uniform plan by each society,—and the returns and tables prepared from them sent to the Smithsonian Institution, where their compilation and arrangement into a more comprehensive form could be effected by a committee of that body."

The great utility of such an arrangement, if carried out, is manifest ; all that we object to it is, that it is *less* useful, and less practicable and certain than the interests of the profession and the community demand. Why should such returns be confined to the cases of *fatal* disease ? These alone may be appropriate to the compilations of the Smithsonian Institute,—but, for the National Medical Association, it would seem desirable to obtain more ample and instructive results. And, again, the mode of distribution of the forms of inquiry appears to us to be incomplete. They are to be furnished by the State Medical Societies to their members. Such forms should, rather, be furnished by every County Society to each of its members, with instructions to return them, with answers, to a committee of the Society. This committee, then, forwarding its report to the State Society's committee, a summary or compilation of returns from all the counties within its domain might be in turn handed to the Committee of the National Association. If such returns, as to the nature, amount and results of prevailing diseases, were thus concentrated at stated periods in the hands of a committee serving the national body for five years, most important data could no doubt be thus obtained, in a shape more capable of accurate generalization than any less extended and systematic research could give. It is probable, also, that no difficulty would exist in the way of obtaining full and regular answers to such definite questions as might be thus uniformly circulated. Method, the subdivision of labor, and the use of the printing press, make many things easy, which, without these aids, might seem unattainable. At any rate, the plan now in vogue having failed, to a considerable extent at least, it would seem a matter of evident expediency to give trial to any other that appears at all likely to succeed.

We will conclude, by merely hinting at the kind of questions, which might form the basis of a circular of inquiry, to be distributed at stated periods (quarterly, for example) by a committee of each county Society, to its members.

1. What diseases have been most prevalent under your observation during the past three months ?

2. Have any *peculiarities* attended the course of the prevalent maladies you have observed during that time ?

3. Has any peculiarity, of type or tendency, affected the course of *ordinary diseases* during that time, within your limits ?

4. Have any *new results of treatment* in disease come under your immediate notice during that period ?

5. What has been the number of deaths in your practice during the past three months ; and from what cause in each case ?

If complete and universal returns were not at first obtained to all these queries, we imagine that more at least would be produced than by the present plan; and the important effect would also follow from it, that practitioners would thus be stimulated and assisted in the task of recording constantly the main items of their observation, and of making them available for the common benefit of the profession and of humanity.

